# Hyperemesis gravidarum induced refeeding syndrome causes blood cell destruction: a case report and literature review

**DOI:** 10.1186/s12884-021-03821-6

**Published:** 2021-05-09

**Authors:** Xiyu Pan, Ran Chu, Jinyu Meng, Qiannan Wang, Yue Zhang, Kun Song, Xingsheng Yang, Beihua Kong

**Affiliations:** Department of Obstetrics Gynecology, Cheeloo College of Medicine, Qilu Hospital, Shandong University, Shandong 250012 Jinan, China

**Keywords:** Hyperemesis gravidarum, Refeeding syndrome, Hypophosphatemia, Intravascular hemolytic anemia, Thrombocytopenia

## Abstract

**Background:**

Hyperemesis gravidarum (HG) is a common complication during pregnancy, however, HG associated simultaneous onset of blood cell destruction due to electrolyte abnormalities is rare. In this case, a woman with refeeding syndrome (RFS) secondary to electrolyte abnormalities caused by severe HG was diagnosed and managed in our hospital.

**Case presentation:**

A 29-year old woman was sent to the local hospitals because of severe HG with appetite loss, weight reduction, general fatigue, and she was identified to have severe electrolyte abnormalities. However, the electrolyte abnormalities were not corrected promptly, and then she had the symptoms of stillbirth, altered mental status, visual hallucination, hemolytic anemia and thrombocytopenia. After transferred to our hospital, we continued to correct the electrolyte abnormalities and the labor induction was performed as soon as possible. The symptoms of blood cell destruction were relieved obviously, and the patient discharged four days later. The electrolyte disturbances and physio-metabolic abnormalities caused by HG helped us diagnose this case as RFS.

**Conclusions:**

This case emphasizes that patients with RFS should be diagnosed appropriately and intervened promptly in order to prevent electrolyte imbalance induced blood cell destruction.

## Background

Although the estimated incidence is 0.3-3 % among all pregnancies, hyperemesis gravidarum (HG) is thought to be the most common cause of hospitalization during the first half of pregnancy in the United States [1]. There is no international consensus on the definition of HG, and it is so far clinically diagnosed after other causes of vomiting and nausea have been excluded [2]. HG-induced excessive vomiting and anorexic condition can result in prolonged starvation, electrolyte abnormalities and may also play an important part in the development of malnourished state [3].

In those severe conditions caused by HG, refeeding syndrome (RFS) can develop rapidly even upon nutritional therapy or under natural oral intake, with the manifestation of severe electrolyte disturbances and physio-metabolic abnormalities [4]. RFS was commonly connected with total parenteral nutrition (TPN), and may acutually occur with all kinds of application modes, including either oral, enteral, or parenteral routes [5]. Identification of high-risk patients is crucial.

Hypophosphatemia, which is the hallmark biochemical feature of RFS [6], is considered as an infrequent cause of intravascular hemolytic anemia. Melvin JD et al. [7] demonstrates a tight relationship between serum phosphate level and red blood cell survival. The study of Yawata and his colleagues [8] emphasizes that hypophosphatemia also causes the platelet broken.

We herein describe an uncommon case of HG followed by RFS-associated hypophosphatemia which then induces blood cell destruction, in order to provide a clinical reference for the accurate diagnosis and prompt treatment of RFS.

## Case presentation

A 29-year old woman, gravida 3, para 2, presented in the 14 4/7 weeks of pregnancy. The current pregnancy was natural conception. She had a history of HG in the first pregnancy. During the current pregnancy, she had been complaining of severe nausea and vomiting for more than a month. In the past 2 weeks, the symptoms became even worse, and she had been unable to eat and drink normally.

**Fig. 1:**
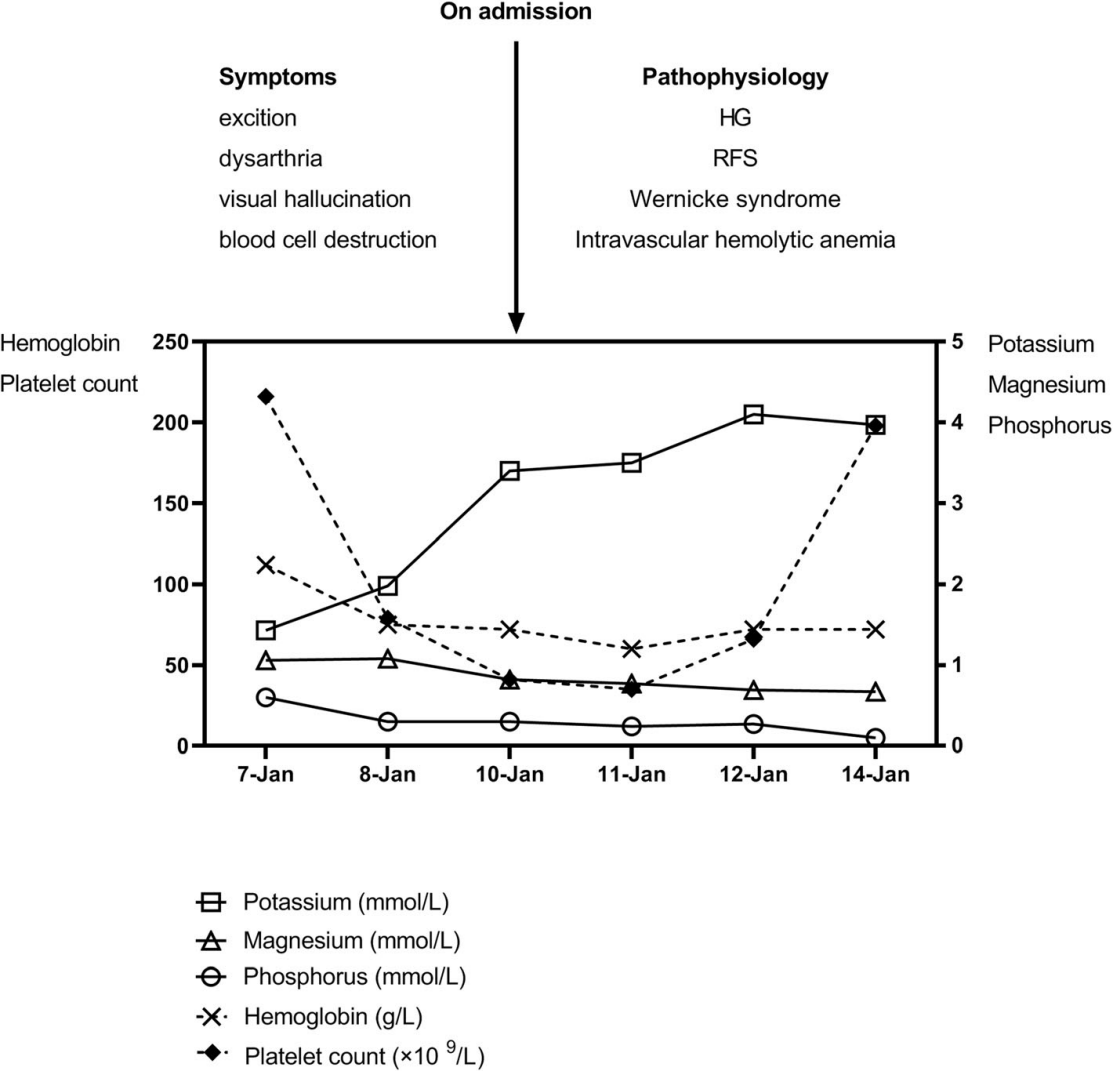
Timeline of the clinical characteristics and laboratory test The upper part of Fig. 1 shows the clinical symptoms and pathophysiology of patient on admission. The lower part shows the biochemical and physiological characteristics of the patient. HG, hyperemesis gravidarum; RFS, refeeding syndrome

Since Jan.7th 2019, the patient was sent to several hospitals successively for treatments. Laboratory tests drawn at the local hospitals (Fig. 1; Table 1) were notable for electrolyte abnormalities, elevated alanine aminotransferase (ALT) level and aspartate aminotransferase (AST) level, as well as the progressive descent of platelet (PLT) count and hemoglobin (HGB) level. The patient accepted fluid replacement therapy, anti-vomiting therapy and nutritional supplement therapy in the local hospitals. However, the treatments did not work, and the disease continued on its course. Apart from nausea and vomiting, new symptoms occurred suddenly, such as decreased mobility, loss of interest, sleeping too much, apathy, dysarthria, blurred vision, and visual hallucination. Two days later, the patient’s mental state changed from apathy to excitation and appeared psychological changes. On Jan. 8th, the fetal death was diagnosed by obstetric ultrasound.

**Table 1:** Laboratory test in the pre and after the labor induction

Laboratory test	Jan. 8th	Jan. 10th	Jan. 11th	Jan.12th	Jan.14th
Potassium level (mmol/L)	1.98	3.40	3.50	4.10	3.97
Magnesium level (mmol/L)	1.08	0.82	0.77	0.69	0.67
Phosphorus level (mmol/L)	0.30	0.30	0.24	0.27	0.10
Chlorinum (mmol/L)	103.50	101.00	104.00	103.00	106.00
Sodium (mmol/L)	140.60	133.00	135.00	134.00	139.00
Calcium (mmol/L)	2.17	2.18	2.08	2.08	2.11
Hemoglobin (g/L)	75.00	72.00	60.00	72.00	72.00
Platelet count (×10^9^ /L)	79.00	41.00	35.00	66.00	198.00
WBC (×10^9^ /L)	11.25	7.90	4.80	9.32	5.13
RBC (×10^9^ /L)	2.47	2.26	1.89	2.44	2.26
RET (%)			0.81		
IRF			29.30		
Hct		19.20	16.10	21.90	20.20
PCT (%)		0.035	0.04	0.093	0.20
HbA1c (%)		5.90			
ESR (mm/h)		32.00			
Glucose (mmol/L)		6.90			
TBIL (µmol/L)	93.70	83.00	57.00	53.00	24.40
IBIL (µmol/L)	25.25	16.00	18.00	22.00	8.30
DBIL (µmol/L)	68.45	28.00	14.00	9.00	16.10
LDH (IU/L)		779.00	790.00		
AST (U/L)	106.00	100.00	55.00	41.00	38.00
ALT(U/L)	183.00	147.00	110.00	83.00	61.00
FT3 (pmol/L)		3.39			
FT4 (pmol/L)		19.74			
TSH (mIU/L)		0.009			
Ketone body		++	+	+	-
CRP (mg/L)		58.83			
Urine volume (ml)		2720.00	1120.00	1900.00	4470.00
Urinary specific gravity		1.021			1.012
BUN (mmol/L)	7.30	3.60	2.70	1.90	1.80
Cr (µmol/L)	42.00	33.00	27.00	24.00	32.00
TP (g/L)		49.00	42.00	44.00	44.40
Albium (g/L)	28.10	22.00	20.00	22.00	27.90
β-HCG (mIU/ml)	182074.30		91231.00		
CK-MB (ng/ml)			1.60		
Ferr (ng/ml)			1472.00		
VitB12 (pg/ml)			158.70		
FOL (ng/ml)			0.70		
PT (s)		14.20	12.90	14.50	12.60
APTT (s)		31.00	25.10	32.10	26.10
Fib (g/L)		4.34	3.48	3.59	2.33
D-Di (µg/ml)		1.28	0.44	1.28	0.46
O_2_ sat (%)		97–100	96–100	97–100	97–99

*WBC* White blood cell, *RBC *Red blood cell, *RET* Reticulocyte, *IRF *Immature reticulocyte fraction, *Hct *Hematocrit, *PCT *Platelet crit, *HbA1c *Glycosylated hemoglobin, *ESR *Erythrocyte sedimentation rate, *TBIL *Total bilirubin in serum, *IBIL *Indirect bilirubin, *DBIL *Direct bilirubin, *LDH *Lactate dehydrogenase, *AST *Aspartate aminotransferase, *ALT *Alanine aminotransferase, *FT3 *Free triiodothyronine, *FT4 *Free thyroxine, *TSH *Thyroid-stimulating hormone, *CRP *C-reactive protein, *BUN *Blood urea nitrogen, *Cr *Creatinine, *TP *Total protein, *β-HCG *β-human chorionic gonadotropin, *CK-MB *Creatinine kinase-MB, *Ferr *Ferritin, *VitB12 *Vitamin B12, *FOL *Folic acid, *PT *Prothrombin time, *APTT *Activated partial thromboplastin time, *Fib *Fibrinogen, *D-Di *D-dimer; *O*_*2*_* sat *Oxygen saturation

The patient was transferred to the Emergency Obstetrics and Gynecology Department in our hospital urgently on Jan. 10th. In the emergency room, laboratory tests showed obviously decreased PLT count (41 × 10^9^/L, normal range: 125–350 × 10^9^/L) and HGB level (72 g/L, normal range: 115-150 g/L), increased serum levels of ALT (147U/L, normal range: 7-40U/L) and AST (100U/L, normal range: 13-35U/L). The patient was also accompanied with the hypophosphatemia (0.30mmol/l, normal range: 0.81-1.45mmol/l) and hypokalaemia (3.4mmol/l, normal range: 3.6–5.0mmol/l). The arterial blood gas was not sampled, and the difference between sodium and chloride could not indicate that she had alkalosis or acidemia. There were no obvious changes in the patient’s electrocardiogram, such as long QTc intervals, flatted T wave or U wave were found. She presented with oliguria and redness in urine, as well as other dehydration symptoms, such as thirsty, dry stools, dry skin and dry mouth. Based on the ketone bodies found in the urine evaluation, the patient was deemed to suffer from a starvation ketosis induced by severe dehydration and metabolic derangements. On examination, the patient had no symptom of bleeding and the vital signs were normal. At her height and weight, she had a BMI of 28.3. The patient still had the symptoms of excitation, dysarthria, and visual hallucination, while the blurred vision receded. An ultrasound scan was performed immediately, which confirmed the absence of fetal cardiac activity.

**Fig. 2:**
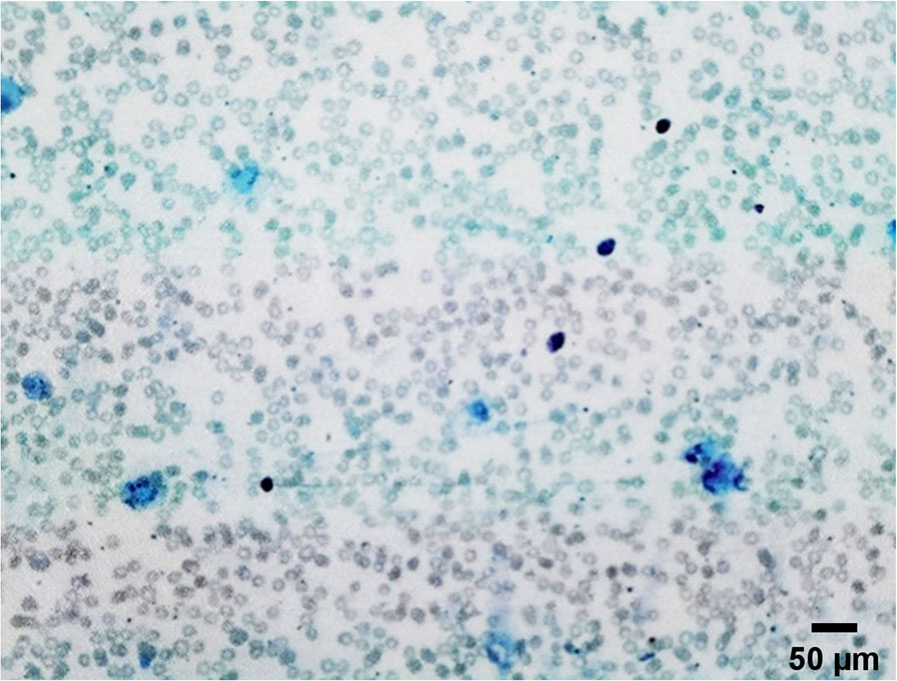
The picture of peripheral blood smear. Figure 2 indicates the picture of peripheral blood smear. HE×100. There was no significant change in the number of white blood cells, and the proportion was generally normal. The red blood cells varied slightly in size, the central light staining area of most red blood cells was slightly enlarged.

The patient was admitted to the obstetrics department of our hospital. Laboratory tests were monitored minutely and shown in Table 1. The patient had no history of paralysis, thyrotoxicosis or use of insulin. Because of nausea and vomiting with an inability to tolerate significant food or drink, standardized commercial parenteral nutrition, hypokalaemia correction (1.0 g of potassium chloride in 500ml of 5 % glucose and 0.9 % sodium chloride solution twice a day) and hypophosphatemia correction were performed. In the context of our patient, the altered mental status was attributed to Wernicke encephalopathy (WE), so treatment with thiamine (200 mg intramuscularly twice a day) was provided immediately and the symptoms started to recover remarkably. According to multidisciplinary consultation shown in Table 2, the patient was diagnosed with intravascular hemolytic anemia. Figure 2 showed the picture of peripheral blood smear.

**Table 2:** Multidisciplinary consultation

Program	Result
Abdominal ultrasound	Normal
Anemia series	Ferritin 1472.00ng/ml, Vitamin B12 158.70pg/ml, folic acid 0.70ng/ml
Cancer series	CYFRA 5.23ng/ml, AFP 44.80ng/ml, ferritin 1392.00ng/ml, CA-125 59.60U/ml
Peripheral blood examination of broken red blood cells	There was no significant change in the number of white blood cells, and the proportion was generally normal. The red blood cells varied slightly in size, the central light staining area of most red blood cells was slightly enlarged. There were no broken red blood cells
Coomb’s test	IgG (-), C3d (-)

*CYFRA *Cytokeratin fraction, *AFP *Alpha-fetoprotein, *CA-125 *Cancer antigen 125, *IgG *Immunoglobulin G

In order to relieve the patient’s symptoms, we decided to induce labor as soon as possible. On account of the severe symptoms of blood cell destruction and hepatic injury which was indicated by the elevated aminotransferase levels, the method of labor induction was chosen cautiously. And water bag was considered as the best choice, because it could avoid the further damage caused by the drug therapy. On January 11th, the water bag was put in her cervix, and she received two units of red blood cell transfusion after that. On the morning of the next day, the delivery was finished. The patient delivered a 60-gram fetus, whose sex was still not identifiable. The routine was complicated by a retained placenta, which required a dilation and curettage. The patient received another two units of red blood cell and one unit of the platelet transfusion followed by the curettage. The immediate blood routine examination after the blood transfusion showed that HGB level was 77 g/L, and PLT count was 86 × 10^9^/L. On the following day, HGB level maintained stability as 72 g/L, and PLT count increased up to 198 × 10^9^/L.

Based on these clinical symptoms, the significant pathophysiological situation can be attributed to RFS on severe malnourished state due to HG, which led to WE, electrolyte abnormalities and eventually resulted in blood cell destruction. The patient’s vomiting persisted until the day of admission, during the period of labor induction, the vomiting stopped. Meanwhile, after the labor induction and symptomatic supportive treatments, laboratory tests showed a gradual normalization of aminotransferase levels, and the patient’s mental state significantly improved, while the symptoms of blood cell destruction started to recover. The patient discharged four days later, and remained in good health during the follow-up period.

## Discussion and conclusion

The reports of blood cell destruction induced by HG are rare, nearly no assays have discussed the mechanism. HG causes acute starvation as well as electrolyte abnormalities, and if they are not rectified correctly, the RFS will occur and develop quickly. Abnormal loss by vomiting, insufficient intake, and previous inappropriate fluid infusion, as well as the development of RFS, may even accelerate the severity of electrolyte abnormalities, especially hypophosphatemia due to HG. And hypophosphatemia is a rare cause of blood cell destruction.

### Hyperemesis gravidarum

HG may result in severe hypovolemia, vitamin deficiency (especially vitamin B1) and substantial decreases in electrolyte (hypophosphatemia, hypokalemia and hypomagnesemia et al.). The patient may develop metabolic acidaemia in severe cases [9, 10]. HG has adverse impacts on maternal quality of life, and can even affect their physical and psychological health [2]. Several articles mention [11, 12] that HG may result in adverse pregnancy and perinatal outcomes. If HG is treated properly, it does not increase the risk of perinatal morbidity or mortality [13]. And maternal death from severe nausea and vomiting of pregnancy is rarely reported nowadays [14]. However, once HG is complicated by WE, nonphysiological termination of pregnancy has been reported in 47.9 % of cases [15]. And electrolyte abnormalities associated with HG have been noted to increase the maternal mortality in some articles [1, 4]. Life-threatening complications such as renal failure, esophageal rupture, and neurological sequelae have also been reported in some intractable cases.

### Refeeding syndrome

The universally accepted definition of RFS is lacking, and it is commonly defined as the potentially fatal shifts in fluids and electrolytes that may occur in malnourished patients receiving artificial nutritional therapy [5, 16]. The clinical features of RFS are very complicated, including vitamin deficiency, electrolyte disturbance as well as changes in nutrients metabolism [17]. The RFS may cause a series of severe complications, including WE, rhabdomyolysis and diabetes insipidus. And blood cell destruction is also a rare complication associated with this syndrome.

The hallmark biochemical feature of RFS is hypophosphatemia [18]. The real incidence of RFS remains unknown, partly owe to the absence of the universally accepted definition. Thus, some scholars adopt the incidence of the hypophosphatemia to replace it. Several prospective and retrospective cohort studies [6, 19, 20] have reported the incidence of hypophosphatemia as 0.43 % in hospitalized patients and up to 48 % in severely malnourished patients who are being refed. The high-risk population mainly contains patients with anorexia nervosa, cancer, uncontrolled diabetes mellitus and alcoholism [21]. However, pregnant women and their foetuses may also be at high risk in some conditions, and they are uniquely vulnerable to the devastating effects of this disease [20].

### Hypophosphatemia

Although hypophosphatemia is ordinarily very unusual in the general population, it can affect 0.43-3 % of hospitalized patients and up to 28 % of intensive care units patients [22]. The study of Mehanna HM et al. [19] establishes that, in patients who receive TPN solutions not containing phosphorus, the incidence of hypophosphatemia can reach up to 100 %. Acute hypophosphatemia with phosphate depletion is common in the hospital setting and can lead to significant morbidity and mortality [23].

Phosphorus predominantly exists in the cell. It plays an important role in all intracellular procedures, structural integrity of cell membranes, and energy storage in the form of adenosine triphosphate (ATP) [5]. Hypophosphatemia results from three main ways: digestive absorption decreasing, kidney excretion rising, and phosphorus transferring to the intracellular compartment [22].

RFS is a rare cause for hypophosphatemia, and it takes effect mainly by the third way. The detailed mechanism is as follows. Once feeding is recovered after a long time of starvation, glycaemia will result in increased secretion of insulin [19]. Under the stimulation of insulin, a large number of phosphate groups move from extracellular spaces into intracellular spaces, in order to be used for the process of phosphorylation and synthesis of ATP. These processes result in a deficiency in intracellular as well as extracellular phosphorus, thus causing the hypophosphatemia [24, 25].

In this case, the following reasons may aggravate the severity of hypophosphatemia due to HG: (1) a decrease in digestive absorption by vomiting, (2) insufficient intake, (3) inappropriate fluid infusion, as well as (4) RFS associated intracellular shift.

### Intravascular hemolytic anemia

Familiar causes of hemolysis include immune-mediated erythrocyte destruction, erythrocyte enzyme defects, microangiopathic hemolytic anemia, toxins and so on [26]. Nevertheless, Melvin JD’s study [7] emphasizes that hypophosphataemiais also an infrequent cause of intravascular hemolytic anemia.

The hypophosphatemia leads to the consumption of red blood cell (RBC) phosphorus as well as the RBC ATP, which is the primary source of energy for RBC function and plays an essential part in the structural integrity. The use up of ATP switches the shape of RBC from a deformable, biconcave disc to a rigid spherocytes or schistocytes shape. The spheroidal, dehydrated, poorly filterable condition shortens the RBCs’ survival time and increases the opportunity of destruction in the microvasculature. So we speculate that hypophosphatemia is likely to influence the function and survival time of RBCs through depletion of cellular ATP and finally results in intravascular hemolytic anemia.

### Thrombocytopenia

Platelets are derived from megakaryocytes, and they play important parts not only in thrombosis and wound repair but also in inflammation, immunity, and cancer biology [27]. There are many different etiologies of thrombocytopenia including nutritional deficiency (such as Vitamin B12, folate) [28] and hypophosphatemia.

Vitamin B12 and folate take effect in platelet production, and their deficiency can cause the depletion of platelet. In this case, the decrease in digestive absorption and insufficient intake caused the patient’s deficiency of Vitamin B12 and folate. However, the deficiency may not be one of the reasons for thrombocytopenia of our patient.

The most important reason for thrombocytopenia in this case is still hypophosphatemia. In the study of Yawata and his colleagues [8], they announce that the hypophosphatemia can result in the ATP depletion in platelets, then clot retraction becomes faulty and leads to a 5- to 10-fold decrease in platelet survival. Thus, hypophosphatemia is a considerably rare cause of thrombocytopenia.

In conclusion, severe HG followed by RFS can be a reason of electrolyte abnormalities such as hypophosphatemia. And this syndrome is always accompanied with a series of severe complications, including blood cell destruction. In clinical practice, if the patients with severe HG get more and more worse after the nutritional therapies and accompanied with PLT and HGB descent, it may be owe to the RFS. The treatments of this syndrome contain aggressive fluid resuscitation, and close monitoring of electrolytes and vitamins in the meanwhile. It’s worth noting that the correct-delivery of fetus and placenta also play an important part in the treatment of RFS syndrome.

## Data Availability

Data are available at Qilu Hospital of Shandong University archives and can be sent by corresponding author on request.

## Abbreviations

HG: Hyperemesis gravidarum
RFS: Refeeding syndrome
ALT: Alanine aminotransferase
AST: Aspartate aminotransferase
PLT: Platelet
HGB: Hemoglobin
TPN: Total parenteral nutrition
WE: Wernicke encephalopathy
ATP: Adenosine triphosphate
RBC: Red blood cell
